# Tetramethylpyrazine blocks TFAM degradation and up-regulates mitochondrial DNA copy number by interacting with TFAM

**DOI:** 10.1042/BSR20170319

**Published:** 2017-05-17

**Authors:** Linhua Lan, Miaomiao Guo, Yong Ai, Fuhong Chen, Ya Zhang, Lei Xia, Dawei Huang, Lili Niu, Ying Zheng, Carolyn K. Suzuki, Yihua Zhang, Yongzhang Liu, Bin Lu

**Affiliations:** 1 Department of Biochemistry, Institute of Biophysics, Attardi Institute of Mitochondrial Biomedicine and Zhejiang Provincial Key Laboratory of Medical Genetics, School of Life Sciences, Wenzhou Medical University, Wenzhou, Zhejiang 325035, China; 2 State Key Laboratory of Natural Medicines, China Pharmaceutical University, Nanjing, Jiangsu 210009, China; 3 Department of Microbiology, Biochemistry and Molecular Genetics, Rutgers University-New Jersey Medical School, Newark, NJ 07103, U.S.A.

**Keywords:** Lon protease, mitochondria, mitochondrial DNA, mitochondrial transcription factor A, Tetramethylpyrazine

## Abstract

The natural small molecule compound: 2,3,5,6-tetramethylpyrazine (TMP), is a major component of the Chinese medicine *Chuanxiong*, which has wide clinical applications in dilating blood vessels, inhibiting platelet aggregation and treating thrombosis. Recent work suggests that TMP is also an antitumour agent. Despite its chemotherapeutic potential, the mechanism(s) underlying TMP action are unknown. Herein, we demonstrate that TMP binds to mitochondrial transcription factor A (TFAM) and blocks its degradation by the mitochondrial Lon protease. TFAM is a key regulator of mtDNA replication, transcription and transmission. Our previous work showed that when TFAM is not bound to DNA, it is rapidly degraded by the ATP-dependent Lon protease, which is essential for mitochondrial proteostasis. In cultured cells, TMP specifically blocks Lon-mediated degradation of TFAM, leading to TFAM accumulation and subsequent up-regulation of mtDNA content in cells with substantially low levels of mtDNA. *In vitro* protease assays show that TMP does not directly inhibit mitochondrial Lon, rather interacts with TFAM and blocks degradation. Pull-down assays show that biotinylated TMP interacts with TFAM. These findings suggest a novel mechanism whereby TMP stabilizes TFAM and confers resistance to Lon-mediated degradation, thereby promoting mtDNA up-regulation in cells with low mtDNA content.

## Introduction

Protein quality control in mitochondria is essential for proper mitochondrial protein homoeostasis (also termed as mitochondrial proteostasis), which maintains mitochondrial function and prevents the proteotoxic stress leading to cellular pathology. Most mitochondrial proteins are located in the matrix where they participate in mitochondrial bioenergetic and metabolic pathways (e.g. tricarboxylic acid (TCA) cycle and  β-oxidation of fatty acids). Mitochondrial matrix contains three ATP-dependent proteases–Lon, caseinolytic peptidase XP (ClpXP) and matrix-oriented AAA (*m*-AAA) [1]. Lon is a nuclear encoded protease, which plays an important role in the quality control of proteins by selectively degrading misfolded or damaged proteins, as well as turning over short-lived regulatory proteins [2,3]. Previous studies suggest that Lon also degrades oxidized proteins [4]. Under endoplasmic reticulum (ER) stress or hypoxia, Lon is up-regulated and may function as a protease as well as a chaperone to facilitate assembly of cytochrome *c* oxidase (COX) complexes that are adapted to low-oxygen conditions [5,6]. The expression level of Lon is important for cell’s fate. In some cell types, down-regulation of Lon impairs mitochondrial function, leads to cell apoptosis and decreases cellular bioenergetics [7–10]. Lon up-regulation has been shown to be tightly correlated with tumorigenesis [8,9,11–15] Taking together, abnormal expression of Lon leading to tumorigenesis has made itself a potential target in the discovery of novel drugs in cancer therapy.

Mitochondrial transcription factor A (TFAM) is a nuclear encoded high mobility group (HMG) protein involved in the replication, transcription and segregation of mtDNA [16–18]. In addition, TFAM plays an important role in the recognition and repair of DNA lesions [17,19,20]. Experiments in *Drosophila* have shown that TFAM is degraded by Lon, thereby down-regulating mtDNA copy number [21]. Our previous work in human cells, demonstrated that when TFAM is not bound to mtDNA, it is rapidly degraded by mitochondrial Lon. [22] However, TFAM that is bound to mtDNA is prevented from degradation. Phosphorylation of TFAM blocks mtDNA binding and promotes its degradation [22]. Depending on the affected tissue, TFAM deficiency decreases mtDNA copy number, leading to respiratory chain disorder and mitochondrial dysfunction [23–25]. Overexpression of TFAM has been shown to ameliorate neuronal death, cardiac mitochondrial dysfunction and ameliorate mitochondrial disease [26–29]. Extracellular signal regulated protein kinases (ERK1/2) reduce mitochondrial transcription by increasing phosphorylation of TFAM, which may have implications for Parkinson’s disease [30]. TFAM may be thus be a novel drug target for the treatment of mitochondria-related diseases.

Given the vital role of Lon to maintain TFAM stability and mtDNA content under mitochondrial dysfunction by proper protein homoeostasis, the finding of specific Lon protease inhibitors or activators will allow us to manipulate the TFAM and mtDNA level by regulating the activity of Lon protease, which will further facilitate the treatment of cancer, as well as neurodegenerative diseases related to protein aggregation in mitochondria. Up to now, only several Lon protease inhibitors have been reported, including 2-cyano-3,12-dioxooleana-1,9(11)-dien-28-oic acid (CDDO) and its derivatives (such as CDDO-Me and CDDO-Im) [12]. CDDO and its derivatives are synthesized based on oleanolic acid and ursolic acid, which have been widely used in traditional chinese medicine as antibacterial, antifungal, anti-inflammatory and anticancer agents for centuries in China. 2,3,5,6-Tetramethylpyrazine (TMP), a natural small molecular compound and one of the major components of Chinese medicine *Chuanxiong*, is widely applied to clinical therapies for dilating blood vessels and inhibiting platelet aggregation and thrombosis. Recent accumulating evidence suggests that TMP protects cardiac function [31]; attenuates inflammation and atherosclerosis development; preserves lens transparency [32]; and reverses multidrug resistance or induces apoptosis in tumour cells [33,34]. In addition, TMP is involved in the protection of oxidative damage of mitochondria caused by reactive oxygen species (ROS), which helps to reduce the mitochondrial dysfunction [35–37].

In the present study, we sought to elucidate the effects of TMP on Lon protease and TFAM. Our results show that TMP blocks the Lon-mediated TFAM degradation and promotes recovery of TFAM expression and mtDNA copy number in HeLa cells with an extremely low mtDNA level; unexpectedly, TMP does not inhibit the activity of Lon protease. Our data suggest that TMP stabilizes TFAM by direct interaction with TFAM, which leads to Lon protease access blocked and thus decreased TFAM degradation.

## Materials and methods

### Cell lines and culture conditions

HCT116 and HeLa ρ^+^ cells were purchased from A.T.C.C. (Manassas, VA). EC-1 cells were obtained from Cell Bank of Shanghai Institute of Biological Sciences, Chinese Academy of Sciences. HeLa cells with an extremely low mtDNA level (HeLa ρ^low^ cells) were established by treating HeLa ρ^+^ cells with ethidium bromide (EB) as described previously [22,38]. HCT116, HeLa ρ^+^ and EC-1 cells were grown in Dulbecco’s Modified Eagle’s medium (DMEM) supplemented with L-glutamine (5 mM), streptomycin (2 μg/ml), penicillin (50 U/ml) and 10% FBS (Life Technologies). HeLa ρ^low^ cells were cultured in the same medium supplemented with sodium pyruvate (110 μg/ml) and uridine (50 μg/ml).

### Reagents

The polyclonal antibody against human Lon synthetic peptide (CRRPQDKDAKGDKDG) was raised in rabbit and affinity purified by GenScript (Nanjing, China). The mouse monoclonal antibody against TFAM was purchased from Abcam (ab176558). The goat polyclonal β-actin antibody was from Santa Cruz (sc-1616). TMP was purchased from Sigma (W323713). Biotinylated TMP was synthesized in our laboratory (see Supplementary data). CDDO was kindly provided by Dr Michael Sporn (Dartmouth University, NH). Bortezomib (Vaecade) was from LC Laboratories (B-1408). All other chemicals used were of highest analytical grade and obtained from Sigma, unless otherwise stated.

### TMP inhibits TFAM degradation by Lon protease *in vitro*

Purified Lon (300 nM monomer) and various concentrations of TMP were incubated for 1 h at 37°C in reaction buffer (50 mM Hepes, 150 mM NaCl, 10 mM MgCl_2_, 0.1 μg/ml BSA) and 1 μM Velcade was used as a positive control. Lon-mediated proteolysis was initiated by the addition of substrate TFAM (150 nM monomer) and ATP (5 mM) and the reactions were incubated for 1 h. Reactions were analysed by Western blot.

### The stability of TFAM^HMG1/2^ and TFAM^wt^ in cultured cells

HeLa ρ^+^ and EC-1 cells were transiently transfected with either TFAM^HMG1/2^ or wild-type TFAM (TFAM^wt^) plasmid using Lipofectamine 2000 (Life Technologies) according to the manufacturer’s instructions. Cells were then seeded in 6-cm dishes 24 h post-transfection. A cycloheximide (CHX) chase was used to block cytosolic protein synthesis; meanwhile, cells were treated with 10 μM TMP for 1, 2 and 4 h. Cellular extracts were prepared to examine the protein level of TFAM^HMG1/2^ and TFAM^wt^ by Western blot analysis.

### Analysis of mtDNA, TFAM and Lon levels in HeLa ρ^low^ cells

The mtDNA depletion–repletion protocol employed in the present study was as follows. Briefly, HeLa ρ^+^ cells were treated with EB (50 ng/ml) for 8 days and then treated with 10 μM TMP upon EB removal for the next 4 days. DMSO worked as a negative control. Cells were harvested at various time points and the protein expression level of Lon, actin and TFAM were analysed by immunoblotting. QPCR was employed to analyse the level of mtDNA. On the other hand, HeLa ρ^low^ cells were treated with different concentrations of TMP for 24 h at 37°C and DMSO worked as a control. Cellular extracts were prepared for Western blot analysis.

### Analysis of human Lon ATPase, peptidase and protease activity *in vitro*

Human Lon was purified as previously described [39]. The inhibitory effect of TMP on Lon ATPase activity was measured using ADP-Glo™ Kinase Assay (Promega) according to manufacturer’s instructions. Lon (300 nM) was pre-incubated for 1 h at 25°C with a series of concentrations of CDDO or TMP. The reaction was initiated after adding 5 mM ultrapure ATP and incubated for 1 h at 25°C after which fluorescence was measured at 620 nm using a Perkin Elmer Victor V and free phosphate was calculated from the standard curve. The method to investigate the inhibitory effect of TMP on Lon peptidase activity was performed as our previous study [12]. Briefly, purified Lon (800 nM monomer) and no enzyme controls were pre-incubated for 1 h at 37°C with various concentrations of TMP in reaction buffer and Velcade was served as a positive control. After adding the fluorescent dipeptide substrate rhodamine110, bis-(CBZ-L-alanyl-L-alanine amide; AA_2_-Rh110, Anaspec; 6 μM) and ATP (4 mM), reactions were initiated and incubated for 3 h at 37°C; the relative fluorescence units (RFUs) were measured at excitation/emission = 485/535 nm by a Perkin Elmer Victor V. Data were analysed by GraphPad Prism 5 software. The inhibitory effect of TMP on Lon protease activity was measured as follows: purified Lon (1 μM) and BSA (0.1 mg/ml) were pre-incubated for 1 h at 37°C with 1 mM TMP in reaction buffer, 5% DMSO worked as a negative control and 1 μM Velcade worked as a positive control respectively. After the additions of ATP (2 mM) and pure casein (60 nM), Lon-mediated degradation of casein was monitored at 30, 60 and 90 min, and the samples were separated by SDS/PAGE (10% gel) followed by staining with Coomassie Brilliant Blue G-250.

### Pull-down using biotinylated TMP

Cell extracts and purified Lon were prepared and mixed with 10 μM biotinylated or non-biotinylated-TMP for 2 h at 4°C, streptavidin (SA)-sepharose was then added into the reaction mixture and incubated for 2 h at 4°C. SA-sepharose was pelleted and washed four times with ice-cold PBS. Immunoblotting was employed to analyse the biotinylated-TMP interacting proteins pulled down by SA-sepharose.

### Quantitative real-time PCR analysis

Genomic DNA was isolated from 1 × 10^5^ cells using TaKaRa MiniBEST Universal Genomic DNA Extraction Kit Ver.4.0 (TAKARA BIO, Shiga, Japan). DNA concentration and purity were checked on the NanoDrop 2000 spectrophotometer (Thermo Scientific, MA, U.S.A.) and all had OD_260_/OD_280_ values of 1.8–2.0. QPCR was performed on Bio–Rad CFX Connect (Bio–Rad, CA, U.S.A.) in a 20 μl volume containing 10 μl SsoFast Probes Supermix (Bio–Rad, CA, U.S.A.), 0.4 μl each primer (10 μM), 0.4 μl probe (10 μM), 2 μl template and 6.8 μl ddH_2_0. The PCR conditions were as follows: initial denaturation at 95°C for 10 s followed by a 40-cycle programme of 95°C for 5 s, 58°C for 30 s. All samples were performed in triplicate with the same conditions, PCR products were analysed by Bio–Rad CFX Manager 2.1 software. We used 2^−ΔΔ*C*^_t_ method to analyse the Q-PCR results. The data represented by six independent experiments, the standard error of S.E.M. was indicated. Sequences of primers and probes are shown in Supplementary data.

## Results

### TMP blocks Lon-mediated TFAM degradation

**Figure 1:**
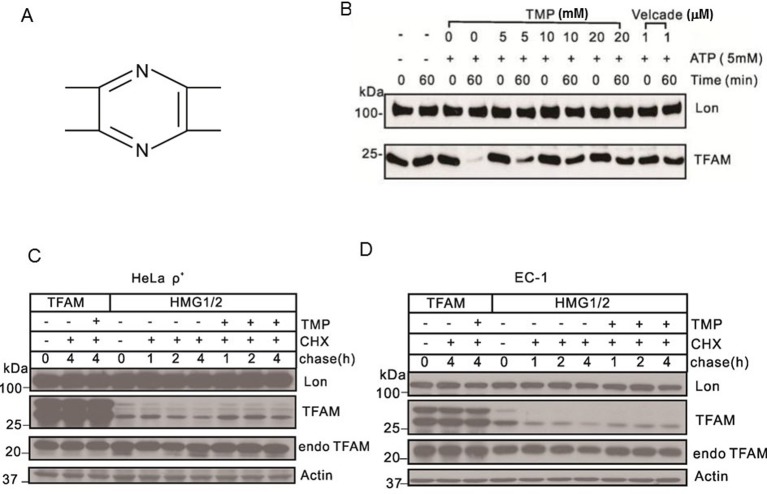
TMP blocks Lon-mediated TFAM degradation (**A**) Chemical structure of TMP. (**B**) Western blot showing levels of Lon and TFAM. Purified Lon (300 nM) was reacted with purified TFAM (150 nM) in the presence of ATP (5 mM) for 1 h. Velcade was used as the positive control. (**C**) Western blot showing levels of Lon, actin and TFAM. HeLa ρ^+^ cells were transfected with plasmids for expressing TFAM^HMG1/2^ or TFAM^wt^ transiently. After transfection, cells were treated with TMP (10 μM) while chased by CHX (100 μg/ml) for the next 4 h. (**D**) Western blot showing levels of Lon, Actin and TFAM. EC-1 cells were transfected with plasmids for expressing TFAM^HMG1/2^ or TFAM transiently. After transfection, cells were treated with TMP (10 μM), while chased by CHX (100 μg/ml) for the next 4 h (endoTFAM, the endogenous TFAM).

To determine the effect of TMP (Figure 1A) on Lon-mediated degradation of TFAM, we analysed the levels of Lon and TFAM in the presence of TMP *in vitro*. We observed that Lon protease rapidly degraded TFAM in less than 60 min in the presence of ATP (Figure 1B). However, the degradation was decreased in a dose-dependent manner in TMP-treated group and was almost completely blocked by TMP at the concentration of 20 mM (Figure 1B).

We next proceeded to analyse the stability of TFAM^wt^ and TFAM^HMG1/2^ in HeLa ρ^+^ and EC-1 cells under the condition of TMP treatment to gain insight into the effect of TMP on Lon-mediated degradation of TFAM in cells. Our previous study demonstrated that if TFAM fails to bind to mtDNA, it will be degraded by Lon [22]. Both TFAM^HMG1/2^ and TFAM^wt^ carry a hexahistidine, which distinguish overexpressed from the endogenous TFAM. Antibodies can recognize the myc-tag fused to mutant or wild-type TFAM. Inactivating mutations in HMG1 and 2 of TFAM were obtained by replacing arginine 157 and lysines 51, 52 and 156 by alanine or glycine. These HMG box replacements impair DNA-binding affinity of TFAM for DNA [40]. In the absence of CHX (T =0), both the precursor and the mature forms of TFAM^HMG1/2^ and TFAM^wt^ were detected in HeLa ρ^+^ cells (Figure 1C). When TMP was absent, the two molecular weight forms of TFAM^HMG1/2^ were degraded. However, when TMP was present, the mature form of TFAM^HMG1/2^ was stabilized (Figure 1C). These results suggested that TMP can protect the mature form of TFAM^HMG1/2^ from degradation by Lon. Both the mature and precursor forms of TFAM^wt^ were very stable even without TMP treatment in HeLa ρ^+^ cells (Figure 1C). Similar results were observed in EC-1 cells (Figure 1D). Taken together, these observations indicated that TMP selectively inhibited Lon-mediated degradation of TFAM in mammalian cells.

### TMP stabilizes TFAM and promotes mtDNA recovery in cells with severe mtDNA deficits

Depletion of mtDNA by treating cells with EB and recovery from this treatment has been employed to study interactions between mtDNA and TFAM [41,42]. Here, we studied the effect of TMP treatment on the recovery of TFAM and mtDNA in HeLa ρ^low^ cells.

**Figure 2:**
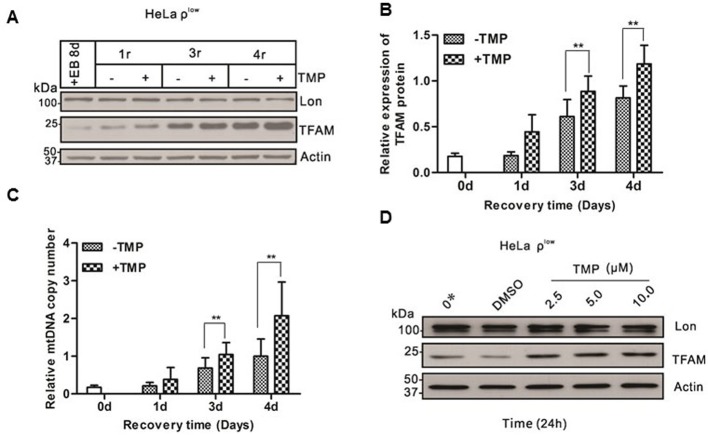
TMP up-regulates TFAM and mtDNA copy number in HeLa cells (**A**) Shown was Western blot analysis of Lon, TFAM and actin from HeLa cells treated with EB for 8 days, cells subsequently grown in the absence of EB (mtDNA recovery) and in the presence of TMP (10 μM) for 1, 3 and 4 days, DMSO (10 μM) was a negative control. (**B**) Quantificative analysis of data from (A) (see ‘Materials and methods’ section). Plotted on the ordinate was the relative expression of TFAM to actin at each time point during the depletion–repletion experiment. Specific time points in the depletion–repletion experiment were labelled as in (A) above. Data represent the mean value ± S.D. from six separate experiments, *P<0.05, **P<0.01. (**C**) Shown was the analysis of mtDNA from the samples described in (A) above. (**D**) Shown was Western blot analysis of Lon, actin and TFAM from HeLa cells with severe mtDNA deficits treated with nothing (lane 1), DMSO (lane 2) or different concentrations of TMP, including 2.5 μM (lane 3), 5 μM (lane 4), 10 μM (lane 5).

MtDNA content of HeLa ρ^+^ cells was depleted to a very low level after EB treatment for 8 days. TFAM was severely reduced concomitantly [42,43] (Supplementary Figures S1A and S1B). TFAM recovered significantly faster in TMP-treated group than in DMSO-treated group during this treatment (Figure 2A,B). Similarly, mtDNA copy number was tightly linked to the TFAM protein level, which was significantly higher in TMP-treated group than that in DMSO-treated group from the third day (Figure 2C). However, the protein level of Lon protease remained unchanged (Figure 2A).

On the other hand, HeLa ρ^low^ cells were treated with various concentrations of TMP. TFAM protein level was increased in a TMP dose-dependent manner, though Lon expression did not change during this treatment (Figure 2D). These results showed that TMP was sufficient to accelerate the recovery of mtDNA and TFAM after EB treatment. However, TMP has no effects to TFAM and mtDNA content in cells with normal mtDNA copy number as shown in Supplementary Figure S4.

### TMP has no inhibitory effect on ATPase, peptidase and protease activity of Lon *in vitro*

**Figure 3:**
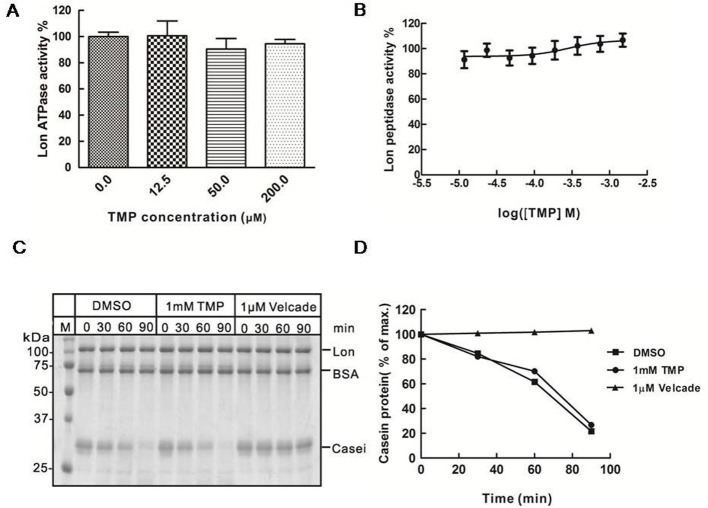
TMP does not inhibit Lon protease activity *in vitro* (**A**) Purified Lon (300 nM) was pre-incubated in the presence of TMP at the indicated concentrations for 1 h at 25°C, after which ATP (5 mM) were added and incubated for 1 h at 25°C before fluorescence values were measured. (**B**) Purified Lon (300 nM) was pre-incubated in the presence of TMP at the indicated concentrations, after which ATP (4 mM) and the AA2-Rh110 (6 μM) were added and incubated for 3 h at 37°C before fluorescence values were measured. (**C**) Coomassie Brilliant Blue staining showing the effect of TMP on Lon-mediated degradation of casein. DMSO worked as a negative control and Velcade worked as a positive control respectively. (**D**) The intensities of reaction bands were analysed with the ImageJ software.

CDDO inhibited the ATPase activity of Lon in a dose-dependent manner (Supplementary Figure S2A), it was used as a positive control. By increasing concentrations of CDDO, the ATPase activity was significantly reduced. The ATPase activity decreased to only approximately 10% when CDDO was 200 μM (Supplementary Figure S2A). As shown in Figure 3A, TMP did not inhibit the ATPase activity of Lon even at the highest concentration of 200 μM we had tested in vitro.

To further explore the effect of TMP on Lon, we tested the peptidase activity by using purified Lon. A fluorescence labelled compound (Z-Ala-Ala) 2-Rh110 (AA_2_-Rh110) was used as a substrate, which does not form complexes with TMP. Lon-mediated cleavage of AA_2_-Rh110 leads to an increase in relative fluorescence. Our results showed that Lon-mediated cleavage of AA_2_-Rh110 was strongly inhibited by Velcade in a dose-dependent manner with an IC_50_ value =28 nM (Supplementary Figure S2B). However, Lon-mediated cleavage of AA_2_-Rh110 was hardly inhibited by TMP (Figure 3B).

We also used native casein as substrate to test the protease activity of Lon. Our data showed that native casein was rapidly degraded by Lon. Although Velcade inhibited the degradation of casein effectively at a concentration of 1 μM, TMP did not block the casein degradation by Lon protease, even at a very high concentration of 1 mM *in vitro* (Figure 3C,D).

Taken together, these results demonstrate that TMP exhibits no inhibitory effect on Lon directly.

### TMP interacts directly with TFAM

**Figure 4:**
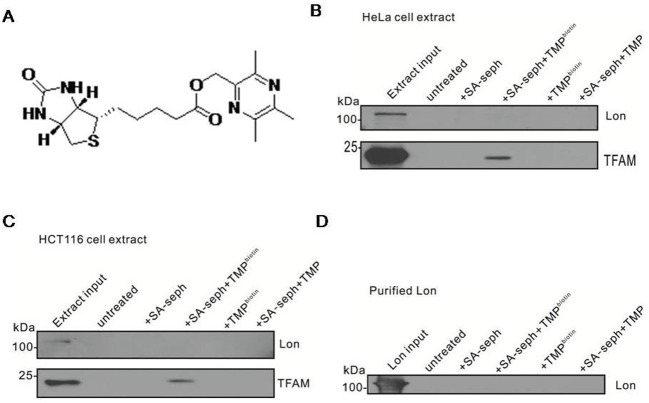
TMP interacts directly with TFAM (**A**) Chemical structure of biotinylated TMP. (**B**) HeLa ρ^+^ cell extracts were reacted with or without TMP or biotinylated-TMP followed by incubation with SA-sepharose, pull-down reactions were immunoblotted with Lon and TFAM. Untreated mock samples contained input but without biotinylated-TMP and SA-sepharose. (**C**) HCT116 cell extracts were reacted with or without TMP or biotinylated-TMP followed by incubating with SA-sepharose, pull-down reactions were immunoblotted with Lon and TFAM. Untreated mock samples contained input but without biotinylated-TMP and SA-sepharose. (**D**) Biotinylated-TMP was reacted with purified Lon and biotinylated-TMP was pulled down with SA-sepharose.

We speculated that TMP may interact with TFAM and/or Lon protease, which leads to the inhibition of TFAM degradation by TMP. To test our hypothesis, we used a biotinylated conjugate of TMP (biotinylated-TMP) (Figure 4A), which has comparable activity with the parent compounds TMP (results not shown) to characterize whether TMP binds Lon or TFAM. Our results showed that TFAM was specifically pulled down only in complexes containing biotinylated-TMP and SA-sepharose, but Lon was hardly detected in HeLa ρ^+^ cells, thereby illustrating a direct interaction between TMP and TFAM (Figure 4B). Similar results were observed in HCT116 cells (Figure 4C). In consideration of a small quantity of Lon proteins in cellular extracts, we used purified Lon to verify the results, it turned out that TMP did not interact with purified Lon (Figure 4D). In conclusion, these findings demonstrate that TMP interacts directly and specifically with TFAM but not with the Lon protease.

## Discussion

Abnormal expression of Lon was associated with various human diseases, such as cancer, aging, MELAS, lipodystrophy and MERRF [36,44,45]. We reported previously that synthetic triterpenoid CDDO and its derivatives can selectively inhibit the ATP-dependent mitochondrial Lon protease and act as either anti-tumorigenic, anti-inflammatory or cytoprotective agent [12]. Thereby, Lon is considered as a potential drug target for prevention and treatment of cancer or other diseases.

Like triterpenoid, TMP is a natural small molecular compound and biosynthesized in plants. It has already been reported that TMP could be used as an antagonist to scavenge ROS for treatment of cardiovascular and cerebrovascular diseases [35]. Recent finding shows that TMP acts as an inhibitor of cyclooxygenase-2 and may have an antitumour effect on non-small cell lung cancer [46]. Moreover, TMP can induce mitochondrial mediated apoptosis in hepatic stellate cells and is considered as a potential choice for the treatment of hepatic fibrosis [34].

We firstly screened out TMP from the chemical database and proposed that TMP may have effects on human Lon or TFAM. Our findings demonstrate that TMP selectively blocks Lon-mediated TFAM degradation *in vitro*, and this supports our initial hypothesis that Lon or TFAM are potential targets of TMP. We confirm that TMP regulates the protein level of TFAM in two ways. First, TMP blocks Lon-mediated TFAM degradation. Our data show that the mature form of TFAM^HMG1/2^ is stabilized in the presence of TMP. However, the precursor and mature form of TFAM^HMG1/2^ is degraded by Lon in the absence of TMP. Interestingly, we also note that TMP treatment exhibits no effect on the level of Lon and TFAM^wt^ in both HeLa ρ^+^ and EC-1 cells (Figure 1C,D). These results are supported by the fact that the DNA-binding affinity of TFAM was impaired by its inactivating mutations in HMG1 and 2, which leads to TFAM degradation by Lon [22]. The fact that TMP blocks Lon-mediated TFAM degradation is further confirmed by the results that purified Lon rapidly degrades purified TFAM *in vitro*; moreover, the degradation is gradually inhibited by TMP in a dose-dependent manner *in vitro* (Figure 1B).

Second, there are a few animal models for studying mitochondrial myopathy, neurodegenerative diseases or apoptosis by disrupting the gene for TFAM [23,47,48]. Previous studies have characterized that Lon protease degrades TFAM to stabilize the TFAM: mtDNA ratio upon reduction in mtDNA copy number in normal cells, so we treated HeLa ρ^+^ cells with EB to establish HeLa ρ^low^ cells, in which TFAM protein was extremely low [49]. We explore the effect of TMP on TFAM and Lon in HeLa ρ^low^ cells. Maintaining an adequate mtDNA content in cells is of great importance [50]. TFAM is likely to be a major regulator of mtDNA copy number, mtDNA copy number changes in parallel with the relative levels of TFAM protein in cultured cells, more specifically, the elevation of TFAM protein results in up-regulation of mtDNA copy number and TFAM down-regulation leads to mtDNA depletion. Our findings indicate that TFAM and the mtDNA copy number are recovered much faster with the treatment of TMP than the control group in HeLa ρ^low^ cells (Figure 2). The results also display that the recovery of mtDNA copy number is delayed in comparison with the recovery of TFAM following TMP treatment. We speculate that there is no effective feedback mechanism to promote mtDNA copy number after TFAM is recovered. In wild-type cells, TFAM is overwhelmingly expressed and binds to mtDNA, which will not be degraded by Lon protease [22]. It is worth mentioning that TMP has little effect on TFAM level or mtDNA copy number (Supplementary Figure S4) in wild-type cells. Our results demonstrate that TMP only inhibits the degradation of TFAM which fails to bind to mtDNA by Lon protease. However, our data (Figure 3A–C) clearly show that TMP has no inhibitory effect on Lon protease. Thus, it is reasonable to consider that TMP may not interact with Lon protease directly.

**Figure 5:**
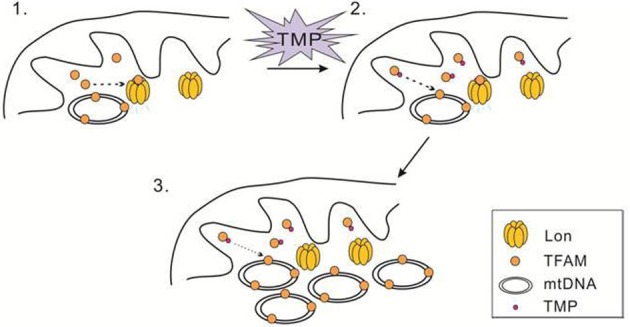
Schematic diagram of TMP blocks Lon-mediated TFAM degradation

TMP ameliorates mitochondrial biogenesis by increasing TFAM, NRF-1 and PGC-1α expression in bEnd.3 and HUVEC cells treated with high glucose [51]. Moreover, TMP also increases these mitochondrial biogenesis related factors in C2C12 myotubes [37]. TN-2 is a novel derivative of TMP, which could protect dopaminergic neurons against MPTP/MPP(+)-induced neurotoxicity possibly by increasing TFAM and activating mitochondrial biogenesis such as PGC-1α and β, indicating that TN-2 may be used to treat PD in future [52]. However, the molecular mechanism responsible for increasing TFAM accumulation by TMP remains unclear. We speculate that TMP may interact with TFAM, which leads the inhibition of TFAM degradation by Lon protease (Figure 5). Our results have shed light on this issue by demonstrating that TFAM is specifically pulled down with biotinylated-TMP (Figure 4B,C), strongly indicating a direct interaction between TFAM and TMP. Based on these results, a conservative presumption is that TMP interacts with TFAM directly to protect TFAM from degrading by Lon. Further experiments are required to determine the way that TMP binding to TFAM. For example, we need to explore if TMP occupies the specific sites of TFAM that are recognized by Lon or changes the structure of TFAM, which leads to TFAM accumulation in the presence of TMP. Since mtDNA and TFAM deficiency is associated with neurodegenerative diseases, heart failure and diabetes, the finding that TMP can increase TFAM and mtDNA level in cells with defects in TFAM and mtDNA contents may have important therapeutic implications for human disease.

## Abbreviations

AAA: ATPases associated with diverse cellular activities
CDDO: 2-cyano-3,12-dioxooleana-1,9(11)-dien-28-oic acid
CHX: cycloheximide
EB: ethidium bromide
HMG: high mobility group
MELAS: mitochondrial encephalomyopathy, lactic acidosis, and stroke-like episodes
MERRF: myoclonic epilepsy with ragged-red fibers
MPP: mitochondrial processing peptidase
MPTP: 1-methyl-4-phenyl-1,2,3,6-tetrahydropyridine
NRF-1: nuclear respiratory factor 1
PD: parkinson's disease
PGC-1a: peroxisome proliferator-activated receptor gamma coactivator 1-alpha
QPCR: quantitative polymerase chain reaction
ROS: reactive oxygen species
SA: streptavidin
TFAM: mitochondrial transcription factor A
TFAM^wt^: wild-type TFAM
TMP: 2,3,5,6-tetramethylpyrazine
